# Correction: Relation between Financial Market Structure and the Real Economy: Comparison between Clustering Methods

**DOI:** 10.1371/journal.pone.0126998

**Published:** 2015-04-20

**Authors:** The PLOS ONE Staff

## Notice of Republication

This article was republished on April 8, 2015, to replace “fi” and “ff” character combinations missing in the Abstract and to correct formatting errors in several equations and figures. The publisher apologizes for the errors. Please download this article again to view the correct version. The originally published, uncorrected article and the republished, corrected article are provided here for reference.

## Supporting Information

S1 File Originally published, uncorrected article. (PDF)

S2 File Republished, corrected article. (PDF)

## References

[1] Musmeci N, Aste T, Di Matteo T (2015) Relation between Financial Market Structure and the Real Economy: Comparison between Clustering Methods. PLoS ONE 10(3): e0116201 doi: 10.1371/journal.pone.0116201 25786703 10.1371/journal.pone.0116201 PMC4365074

