# Supplementary material for: Relation between Financial Market Structure and the Real Economy: Comparison between Clustering Methods
Source: PLoS One. 2015 Mar 18;10(3):e0116201. doi: 10.1371/journal.pone.0116201 (PMC4365074; doi:10.1371/journal.pone.0116201)
Supplement: S1 File — The Supporting Information, S1 File, contains further analysis, some extra details about the methodology and some extra results. (PDF) [file pone.0116201.s001.pdf]

Supporting information for: Relation between financial market structure and the real economy: comparison between clustering methods

Nicoló Musmeci[1], Tomaso Aste[2,3,*], T. Di Matteo[1]
1 Department of Mathematics, King's College London, The Strand, London, WC2R 2LS
2 Department of Computer Science, UCL, Gower Street, London, WC1E 6BT, UK
3 Systemic Risk Centre, London School of Economics and Political Sciences, London, WC2A2AE, UK
∗ **E-mail: t.aste@ucl.ac.uk**

S1 Dataset analysis

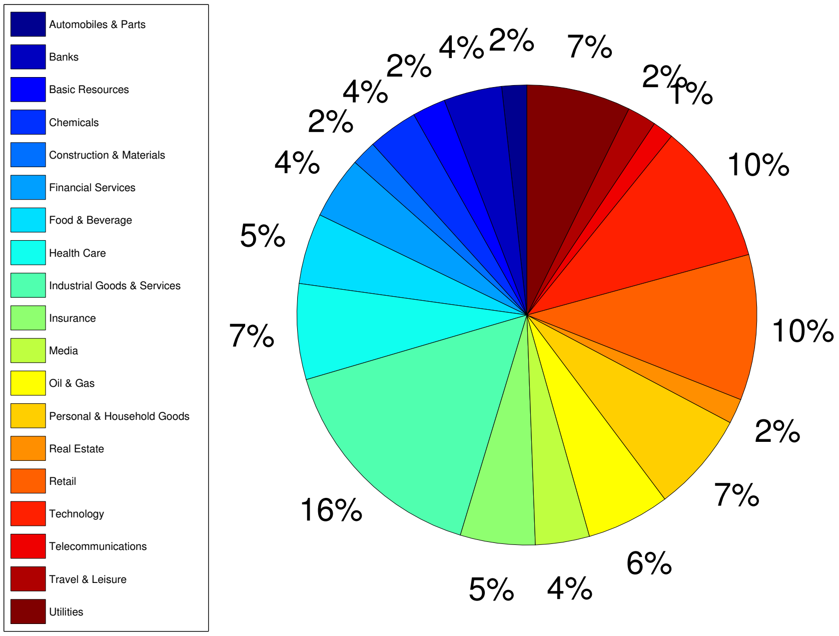

Figure S1. Pie chart showing the composition of the entire set of stocks in terms of ICB supersectors.

The set of stocks has been chosen in order to provide a significant sample of the different industrial sectors in the market. We have chosen the ICB industrial classification, that yields 19 different Supersectors, that in turns gather in 10 Industries: the percentage of stocks belonging to each ICB supersectors is reported in Fig. S1 .

In Fig. S2 two plots are shown that summarize the main features of this dataset. The graphs show the average price $\bar{P}(t) \equiv \frac{1}{N}\sum_i P_i(t)$ and the average log return of the prices, $\bar{r}(t) \equiv \frac{1}{N}\sum_i r_i(t)$, as a function of time. From these plots we can see that both the internet bubble bursting (2002) and the credit crunch (2007-08) are displayed by the market dynamics. In particular it is evident a steep increase in volatility for both periods, strongly autocorrelated in time: a well known feature of log-returns dynamics [1]. Such clusters of volatility can be observed also after the credit crunch, in 2010 and 2012.

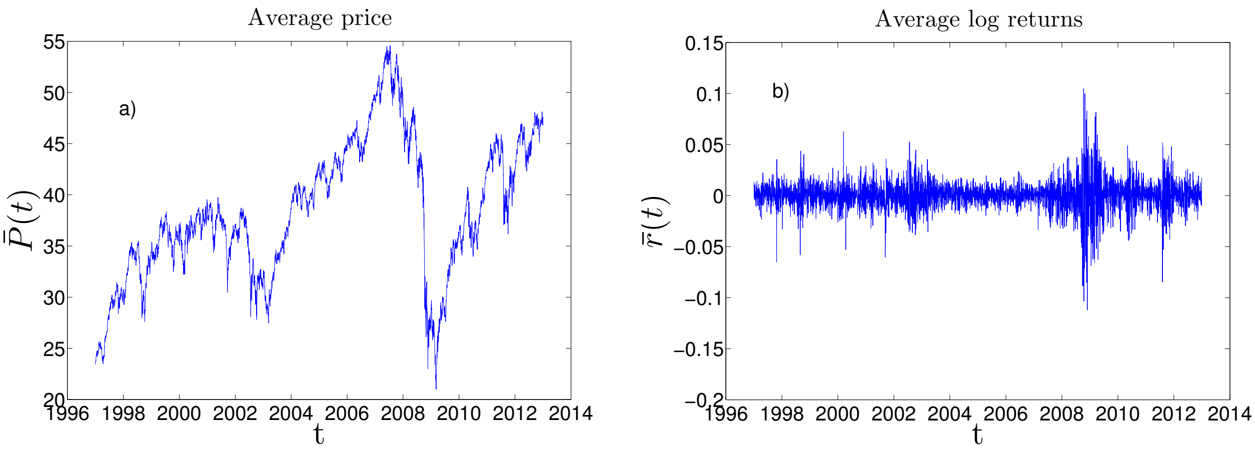

Figure S2. Average price and log-returns of the dataset, from January 1997 to December 2012. a) Average price $\bar{P}(t)$ of the 342 US stocks in the dataset; b) Average log-return $\bar{r}(t)$ of the same prices.

S2 Clustering methods: a brief review

Our focus is on the hierarchical structures (dendrograms) that can best describe the interdependencies in the market, together with a cluster characterization of this structure. To this purpose, the first step is to define a suitable distance between pairs of stocks, knowing the correlation between them. An appropriate function [2] is $D_{ij} = \sqrt{2(1 - C_{ij})}$: it can be shown that with this choice D_{ij} satisfies the three properties of a distance measure.

We then end up with a set of $N \times N$ distance matrices $D(t_k)$ and $D^R(t_k)$, to which we apply two different, well known tools in order to reveal the hidden (unknown) structure of dependencies:

- **Single Linkage** (SL), that is an hierarchical clustering algorithm. Given the distance matrix, it starts assigning to each objects its own cluster, and then at each step merges the closest (i.e. least distant) pairs of clusters into one new cluster, until only one cluster remains. The distances among two generic clusters A and B is everytime defined and updated according to the formula

$$d_{A,B} = \min_{a \in A, b \in B} D(a, b) \qquad (1)$$

  SL is called an *agglomerative* clustering, since it begins with a partition of N clusters and then proceed merging them. The final output of the method is a dendrogram, that is a tree showing the hierarchical structure found by the SL. The distance measure defined in this dendrogram is an ultrametric distance [2]. A proper cluster partition of the stocks can be obtained by choosing the number of clusters (that is therefore a free parameter) and cutting the dendrogram at the appropriate level.

  This algorithm is strictly related to the one that provides a Minimum Spanning Tree (MST) given the distance matrix D. The MST is a tree graph having the stocks as nodes, and it has been used as topological tool in Econophysics since the work of Mantegna [2]. It can be generated starting with an empty graph: after sorting all the correlations in C in descending order, add a weighted link between the two stocks/nodes with the highest correlation, and then go ahead with the next

highest pair correlation; whenever the new link to add generates a loop, do not add that link and skip to the next one, until all the list is checked.

This tree contains exactly $N-1$ links. It can be shown [3] that the MST algorithm is basically the SL procedure carried out until the graph is completely connected. There is therefore a strict relation between the two tools. However the MST retains some information that the SL dendrogram throws away [3] .

- **Average Linkage** (AL) is a hierarchical clustering algorithm similar to SL. The algorithm is the same as the one underlying the SL, but with Eq. 3 replaced by:

$$d_{A,B} = mean_{a \in A, b \in B} D(a,b) \tag{2}$$

- **Complete Linkage** (CL) is a third variant of SL, where Eq. 3 is replaced by:

$$d_{A,B} = \max_{a \in A, b \in B} D(a,b) \tag{3}$$

- **Directed Bubble Hierarchical Tree** (DBHT) [4], a novel hierarchical clustering method that exploits the topological property of the PMFG (Planar Maximally Filtered Graph) in order to find the clustering.

  The PMFG is a generalization of the MST, that is included in the PMFG as a subgraph. It is constructed following the same procedure of the MST, except that the non loop condition is replaced with the weaker condition of planarity (i.e. each added link must not cut a pre-existent link). Thanks to this more relaxed topological constraint the PMFG is able to retain a larger amount of link, and then information, than the MST. In particular it can be shown that each PMFG contains exactly $3(N-2)$ links.

  The basic elements of a PMFG are three-cliques, subgraphs made of three nodes all reciprocally connected (i.e., triangles). The DBHT exploits this topological structure, and in particular the distinction between separating and non-separating three-cliques, to identify a clustering partition of all the nodes in the PMFG [4] . A complete hierarchical structure (dendrogram) is then obtained both inter-clusters and intra-clusters by following a traditional agglomerative clustering procedure.

  The Linkage algorithms look at the sorted list of distances d_{ij} and build the dendrogram by gathering subsets of stocks with lowest distances; the clustering is then obtained, as we said, from the dendrogram after choosing the parameter "number of clusters". The DBHT instead reverses this order: first of all the clusters are identified by means of topological considerations on the planar graph, then the hierarchy is constructed both inter-clusters and intra-clusters. The difference involves therefore both the kind of information exploited and the methodological approach.

- **k-medoids** is a partitioning clustering method closely related to k-means [5]. It takes the number of clusters N_{cl} as an input. The algorithm is the so called Partitioning Around Medoids (PAM), and is as follows:

  1. select randomly N_{cl} "medoids" among the N elements;
  2. assign each element to the closest medoid;
  3. for each medoid, replace the medoid with each point assigned to it and calculate the cost of each configuration;
  4. choose the configuration with the lowest cost;
  5. repeat 2)-4) until no change occurs.

  This method, alike the others taken into account here, is not a hierarchical method and does not provide therefore a dendrogram but only a partition.

S3 Clustering compositions: non-detrended case

DBHT clusters composition

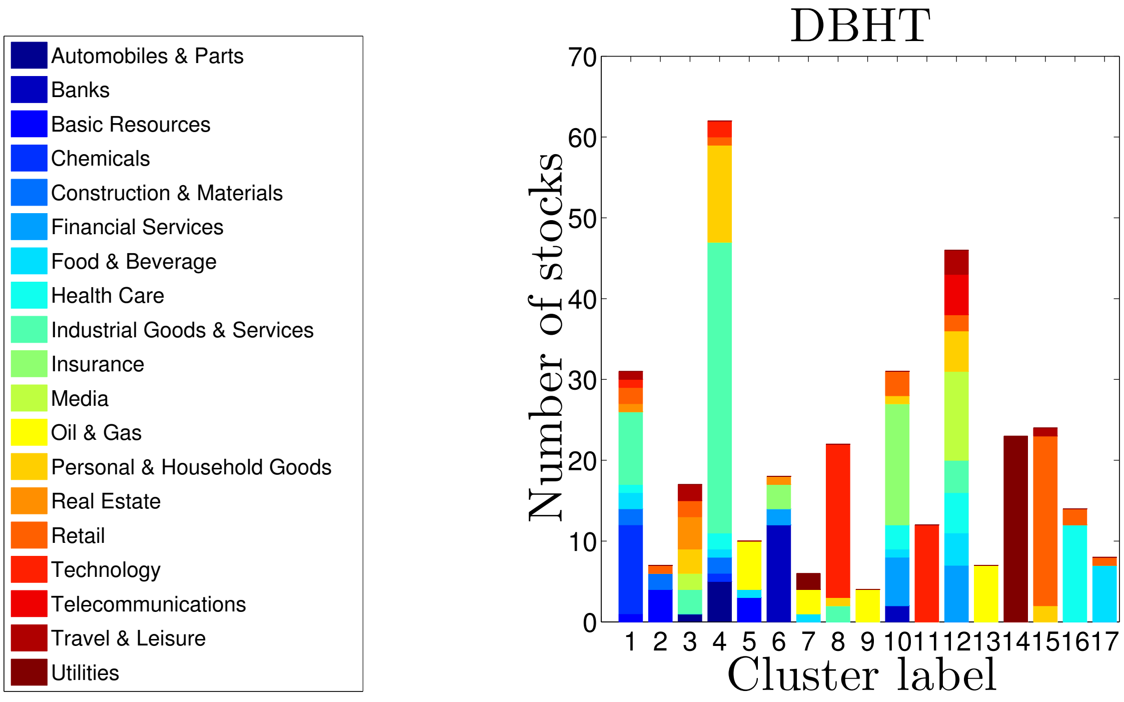

Figure S3. Number of stocks and composition of DBHT clusters in terms of ICB supersectors, on non-detrended log-returns. The composition is shown by using different colours.

In Fig. S3 we report a graphical summary of the clusters obtained applying the DBHT method to the whole time window of data (1997-2012), by using non-detrended log-returns.

The DBHT returns a number of clusters, N_{cl}, equal to 17. Cluster 4, the largest, is made of 62 stocks, accounting for about the 18% of the total number of stocks; cluster 9, the smallest, contains 4 stocks. The average size of clusters is 20.1 stocks. As we can see, four clusters show a composition of stocks belonging to only one ICB supersector : cluster 9 and 13 (Oil & Gas), 11 (Technology) and 14 (Utilities). Similar cases are cluster 8, made of Technology stocks for more than 86%, cluster 15, within which 91% of stocks are from Retail, cluster 16 (75% of stocks from Health Care) and cluster 17 (87.5% of stocks from Food & Beverage). Moreover there are clusters that, although showing a mixed composition, are composed by supersectors strictly related: the number 6 is made of Banks, Financial Services and Insurance, all supersectors that the ICB gathers in the same industry (Financial) at the superior hierarchical step.

There are clusters that do not show an overexpression for a particular supersector or industry: this fact points out that the clustering is after all providing an information that cannot be reduced only to the industrial classification. In particular clusters 1, 3 and 12 have a heterogeneous composition, covering almost all the 19 supersectors and with no sector dominating the others. The cluster 4 is an intermediate case, since even though it overexpresses the Industrial Goods & Services (75%), it contains stocks belonging to 9 different supersectors. Interestingly the largest clusters (4, 12, 1 and 10) are all among these types of "mixed" clusters.

Other clustering compositions

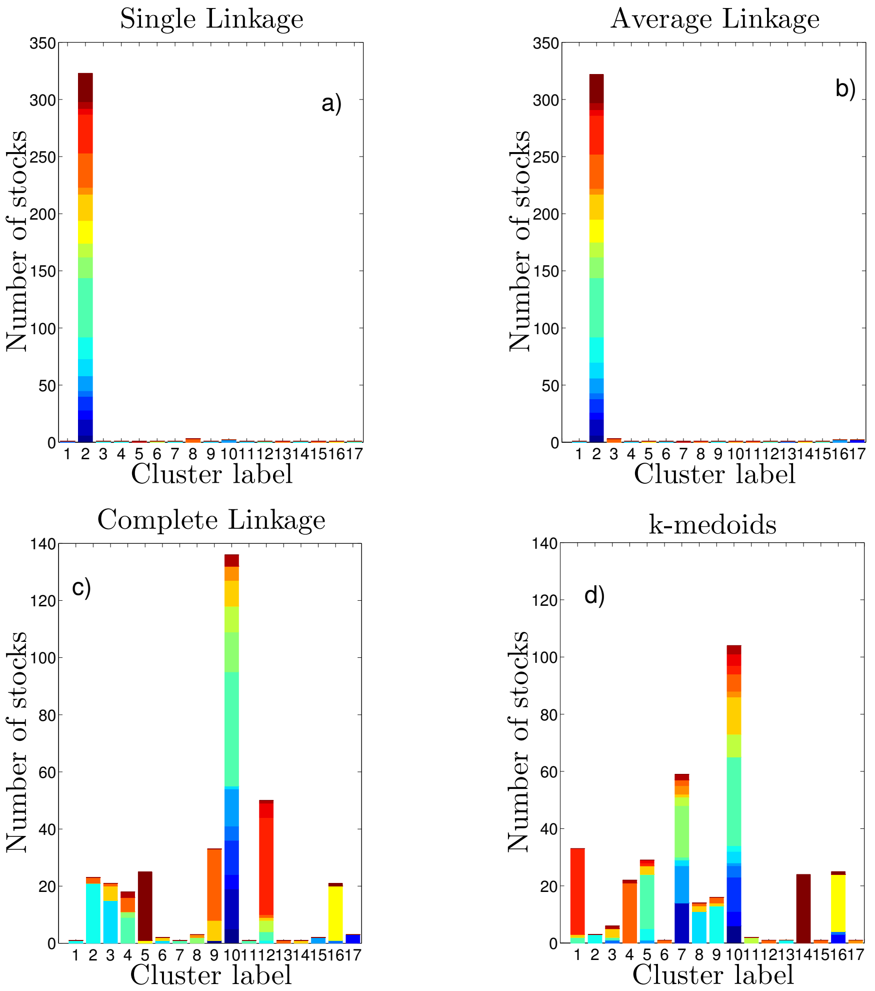

Figure S4. Composition of clustering in terms of ICB supersectors, for different clustering methods, on non-detrended log-returns. The x-axis represents the single cluster labels, the y-axis the number of stocks in each cluster. Each colour corresponds to an ICB supersector (the legend is the same as in Fig. S3. The graphs show the results for a) SL clustering, b) for AL, c) for CL and d) for k-medoids.

We here apply other clustering methods on the same data and compare results with DBHT clustering. The clustering methods considered are Single Linkage (SL), Average Linkage (AL), Complete Linkage (CL) and k-medoids. The latter is not a hierarchical clustering method, so it does not provide a dendrogram: however we analysed it to compare our results with a well established clustering method. The number of clusters, that unlike the DBHT, is a free-parameter for these methods, has been chosen equal to 17 in these cases, in order to compare the bar graphs with the Fig. S3 for DBHT. We plot in Fig. S4 a), b), c) and d) the clusters compositions obtained by using these four clustering methods, namely SL, AL, CL and k-medoids.

First of all we can observe that for each of them there is a strong heterogeneity in the size of clusters: SL and AL display two huge clusters of 323 and 322 stocks respectively (almost identical, having 318 stocks in common), with the other clusters made of one, two or three stocks. For both the algorithms this giant cluster contains stocks of all ICB sectors.

For the CL and the k-medoids the situation is quite different. For the CL, the giant cluster (cluster number 10) is much reduced in size (136 stocks), with also other three clusters (the number 12, 9 and 5) containing a relevant number of stocks (50, 33 and 25 respectively): the main supersectors that are overexpressed are Technology (cluster 12), Utilities (cluster 5), Retail (cluster 9), Oil & Gas (cluster 16) and Health Care (cluster 2). A very similar structure occurs with the k-medoids, but with the giant cluster splitting further in two large clusters (7 and 10). However the DBHT clustering is the one showing the largest degree of homogeneity in size and overexpression of ICB supersectors, at least for this number of clusters (see Fig. S3 for comparison).

Comparing these results with the same analyses on detrended log-returns (Fig. 3 and 4 in the paper) we can conclude that the subtraction of the market mode makes all the clusterings methods (with the exception of SL) more homogenous in size and more able to retrieve the ICB partition. The SL clustering instead does not seem to be sensitive to this subtraction, and keeps not overexpressing any ICB supersector even in the detrended case (Fig. 4 a)).

S4 Bootstrapping test of robustness

The basic idea of the Bootstrapping technique is the following [6]: suppose, for a given time window of length L, we have N time series (one for each stock), each one having length L . We can fit this data in a $N \times L$ matrix, say X, and calculate the correlation matrix for it, say ρ , and a clustering using the DBHT, say Y. Now let us create a replica X' of the matrix X, such that each row of X' is drawn randomly among the rows of X, allowing multiple drawings of the same rows. From X' we can again calculate a correlation matrix ρ' and a clustering Y'.

By repeating this procedure n_{boot} times, we end up with n_{boot} replica of clusterings, each one slightly different from the original one due to the differences between X and its replicas. This sample of replicas can be used to test the robustness of any quantity measured in the original clustering Y, e.g. the number of clusters. This can be done by checking whether the original measure is compatible with the distribution of replicas, performing e.g. a statistical hypothesis test.

References

1. Cont R (2001) Empirical properties of asset returns: stylized facts and statistical issues. Quantitative Finance 1: 223-236.

2. Mantegna RN (1999) Hierarchical structure in financial markets. Eur Phys J B 11: 193.

3. Tumminello M, Lillo F, Mantegna RN (2010) Correlation, hierarchies, and networks in financial markets. J Econ Behav Organ 75: 40-58.

4. Song WM, Di Matteo T, Aste T (2012) Hierarchical information clustering by means of topologically embedded graphs. PLoS ONE 7: e31929.

5. MacQueen J (1967) Some methods for classification and analysis of multivariate observations. Proceedings of the Fifth Berkeley Symposium on Mathematical Statistics and Probability 1: 281-297.

6. Efron B (1979) Bootstrap methods: Another look at the jackknife. Ann Stat 7: 1-26.

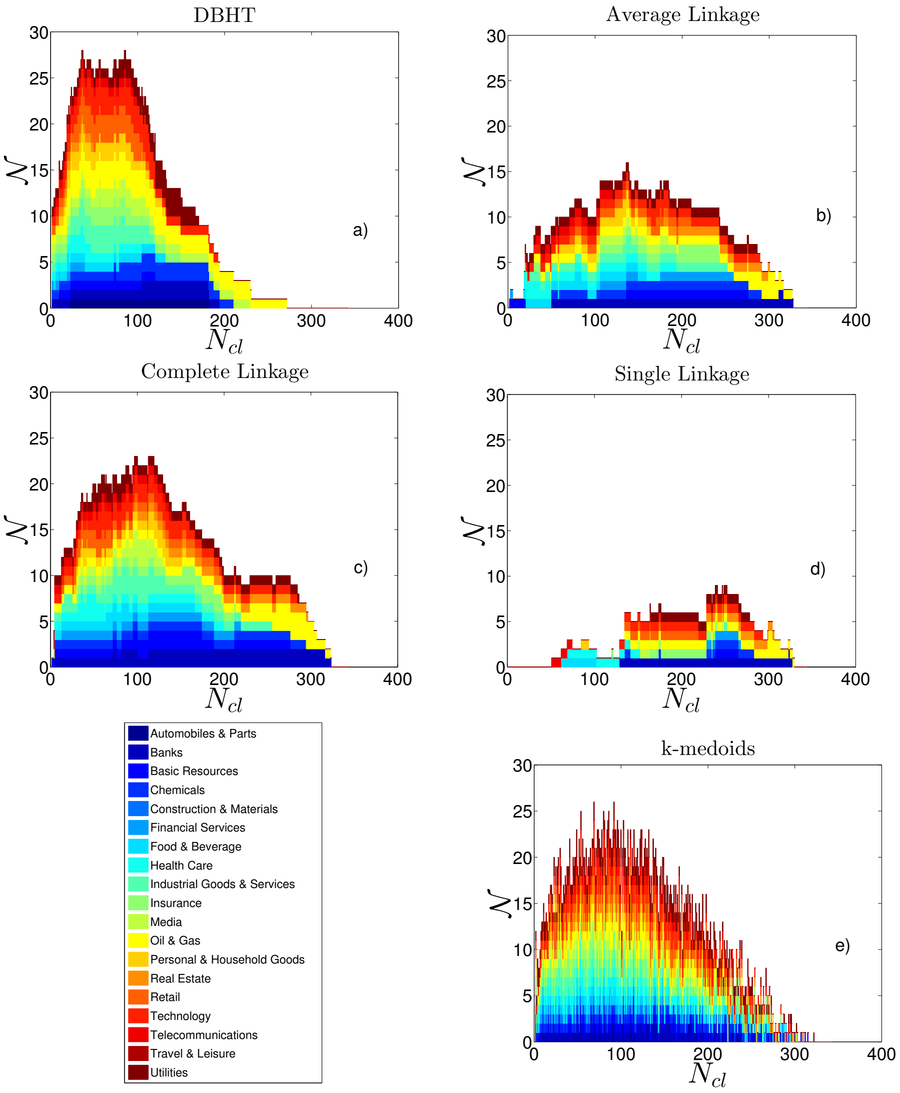

Figure S5. ICB supersectors overexpression at different levels of the hierarchies. Each bar graph shows, varying the number of clusters N_{cl}, how many times ($\mathcal{N}$) an ICB supersector is overexpressed by a cluster, according to the Hypergeometric hypothesis test (i.e., number of tests being rejected). Each colour shows the number of overexpressions for each ICB supersector. In graphs a)-e) the results for DBHT, AL, CL, SL and k-medoids clustering respectively are shown. The correlations are calculated on non-detrended log-returns.

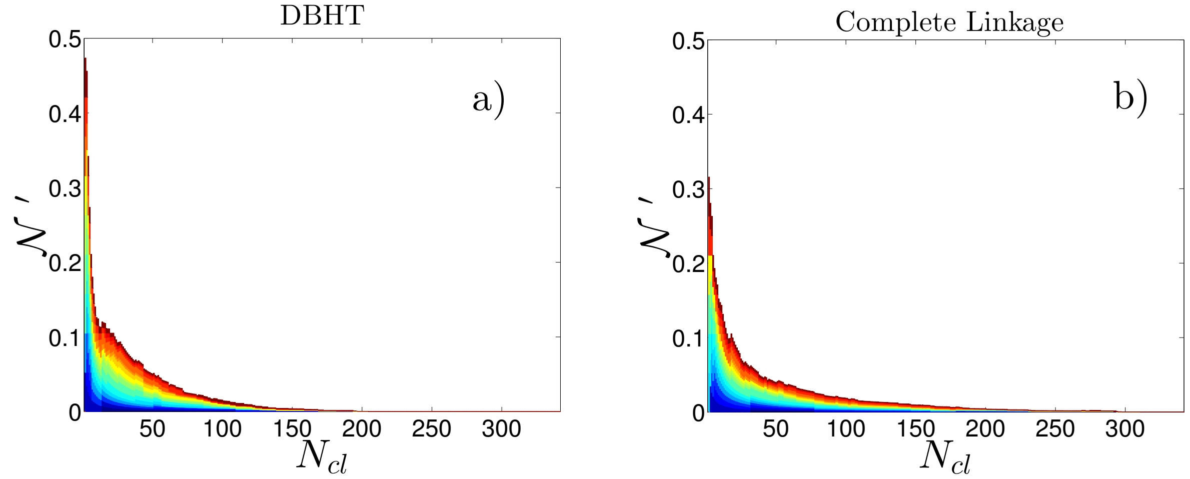

Figure S6. ICB supersectors overexpression as percentage of pairs cluster/supersector rejecting the hypergeometric test. Each bar graph shows, varying the number of clusters N_{cl}, how many times ($\mathcal{N}$) an ICB supersector is overexpressed by a cluster according to the Hypergeometric hypothesis test (i.e., number of tests being rejected), divided by the total number of Hypergeometric tests performed ($0.5 \times N_{cl} \times N_{ICB}$, with N_{ICB} the number of ICB supersectors): $\mathcal{N}' = \frac{2\mathcal{N}}{N_{cl} \times N_{ICB}}$. Each colour shows the number of overexpressions for each ICB supersector. In graphs a)-b) the results for DBHT and CL clusterings respectively are shown. The correlations are calculated on detrended log-returns.
